# Allele-specific transcriptional elongation regulates monoallelic expression of the *IGF2BP1 *gene

**DOI:** 10.1186/1756-8935-4-14

**Published:** 2011-08-03

**Authors:** Brandon J Thomas, Eric D Rubio, Niklas Krumm, Pilib Ó Broin, Karol Bomsztyk, Piri Welcsh, John M Greally, Aaron A Golden, Anton Krumm

**Affiliations:** 1 Institute for Stem Cell and Regenerative Medicine, University of Washington School of Medicine, 815 Mercer St., Seattle, WA 98109, USA; 2 Department of Genome Sciences, University of Washington, 3720 15th Ave NE, Seattle, WA 98195, USA; 3 National Centre for Biomedical Engineering Science, National University of Ireland, Galway, University Road, Galway, Republic of Ireland; 4 Department of Medicine (Endocrinology), Albert Einstein College of Medicine, 1300 Morris Park Ave, Bronx, NY 10461, USA; 5 UW Medicine, South Lake Union, University of Washington School of Medicine, 815 Mercer St., Seattle, WA 98109, USA; 6 Division of Medical Genetics, Department of Medicine, University of Washington, 1705 NE Pacific St., Seattle, WA 98195, USA; 7 Department of Genetics, Albert Einstein College of Medicine, 1300 Morris Park Ave, Bronx, NY 10461, USA; 8 Department of Medicine (Hematology), Albert Einstein College of Medicine, 1300 Morris Park Ave, Bronx, NY 10461, USA; 9 Department of Radiation Oncology, University of Washington, 1959 NE Pacific St., Seattle, WA 98195, USA

## Abstract

**Background:**

Random monoallelic expression contributes to phenotypic variation of cells and organisms. However, the epigenetic mechanisms by which individual alleles are randomly selected for expression are not known. Taking cues from chromatin signatures at imprinted gene loci such as the insulin-like growth factor 2 gene 2 (*IGF2*), we evaluated the contribution of CTCF, a zinc finger protein required for parent-of-origin-specific expression of the *IGF2 *gene, as well as a role for allele-specific association with DNA methylation, histone modification and RNA polymerase II.

**Results:**

Using array-based chromatin immunoprecipitation, we identified 293 genomic loci that are associated with both CTCF and histone H3 trimethylated at lysine 9 (H3K9me3). A comparison of their genomic positions with those of previously published monoallelically expressed genes revealed no significant overlap between allele-specifically expressed genes and colocalized CTCF/H3K9me3. To analyze the contributions of CTCF and H3K9me3 to gene regulation in more detail, we focused on the monoallelically expressed *IGF2BP1 *gene. *In vitro *binding assays using the CTCF target motif at the *IGF2BP1 *gene, as well as allele-specific analysis of cytosine methylation and CTCF binding, revealed that CTCF does not regulate mono- or biallelic *IGF2BP1 *expression. Surprisingly, we found that RNA polymerase II is detected on both the maternal and paternal alleles in B lymphoblasts that express *IGF2BP1 *primarily from one allele. Thus, allele-specific control of RNA polymerase II elongation regulates the allelic bias of *IGF2BP1 *gene expression.

**Conclusions:**

Colocalization of CTCF and H3K9me3 does not represent a reliable chromatin signature indicative of monoallelic expression. Moreover, association of individual alleles with both active (H3K4me3) and silent (H3K27me3) chromatin modifications (allelic bivalent chromatin) or with RNA polymerase II also fails to identify monoallelically expressed gene loci. The selection of individual alleles for expression occurs in part during transcription elongation.

## Background

Allele-specific gene expression is an integral component of cellular programming and development and contributes to the diversity of cellular phenotypes [1,2]. Allelic differences in gene expression are mediated by either parent-of-origin-specific selection (imprinting) or stochastic selection of alleles for activation and/or silencing. The importance of genomic imprinting has recently been highlighted by RNA sequencing studies that demonstrated widespread allelic differences in gene expression in mouse brain affecting more than 1,300 genes [3]. The extent of sex- and stage-specific expression of individual alleles emphasizes the essential role of allelic transcriptional regulation in development. In addition to the extensive occurrence of imprinted parent-of-origin-specific expression, gene expression patterns of clonal cell populations are also modified by random or stochastic silencing of either the maternal or paternal allele. Well-known loci displaying allele-specific expression include odorant receptor genes, immunoglobulins and various receptor proteins [4-6]. Additionally, previous large-scale studies have provided new data demonstrating that parent-of-origin-specific expression is employed much more frequently than previously thought [7]. These new findings illustrate the scale and complexity of genomic allele-specific expression. However, the precise molecular mechanism underlying the allelic bias in gene expression is not very well understood.

The best-characterized locus with strict monoallelic imprinted gene expression is the region containing the insulin-like growth factor 2 (*IGF2*) and *H19 *genes [8]. The regulation of this locus relies on the imprinting control region (ICR), which acquires DNA methylation on the paternal allele during normal development of the male germline. Methylation of cytosines at the ICR inhibits binding of the zinc finger protein CTCF to the paternal allele, preventing its role as an insulator and allowing long-range interactions of the *IGF2 *promoter with enhancer elements downstream of the *H19 *gene [9-11]. In contrast, the unmethylated ICR on the maternal allele recruits CTCF, effectively preventing promoter-enhancer interactions and maintaining repression of the maternal *IGF2 *gene.

The well-documented requirement of CTCF for imprinted expression at the *IGF2*/*H19 *gene locus is thought to result from its role in establishing and/or maintaining long-distance interactions between regulatory elements [12]. Allele-specific binding of CTCF to the ICR has long been known to be essential for the formation of chromatin loops. While the precise mechanism of CTCF's role in long-distance chromatin interactions remains unknown, several studies have provided a rationale for the differential expression of the maternal and paternal *IGF2 *gene by revealing an interaction of CTCF with cohesin, a protein complex known for its requirement during sister chromatid cohesion in mitosis [13-16]. Chromosome conformation capture experiments in combination with RNA interference assays recently confirmed the CTCF and cohesin-dependent formation of higher-order chromatin structures at the *IGF2*/*H19 *and other gene loci [17-19].

In addition to DNA methylation, histone modifications also contribute to the maintenance of allele-specific expression. DNA methylation of ICRs is accompanied by repressive histone markers, including histone H3 trimethylated at lysine 9 (H3K9me3). In contrast, the unmethylated allele is characterized by permissive histone markers, including histone H3 trimethylated at lysine 4 [20]. Colocalization of epigenetic markers including DNA methylation and histone H3 dimethylated at lysine 9 has been exploited to identify epigenetically distinct parental alleles. Chromosomal regions displaying overlaps of euchromatin and heterochromatin-specific markers have been enriched for known imprinted genes [21].

Despite the importance of monoallelic expression in cellular development and differentiation, little is known about the establishment and maintenance of random monoallelic expression. The link between allele-specific binding of CTCF and monoallelic expression of the *IGF2 *gene prompted us to test whether the presence of CTCF and H3K9me3 specifies a chromatin arrangement which demarcates random monoallelically expressed alleles. Using array-based chromatin immunoprecipitation (ChIP-chip), we identified 293 loci displaying these chromatin markers. We selected the *IGF2BP1 *gene locus to further examine whether the presence of CTCF and H3K9me3 comprises a necessary chromatin arrangement for a specific expression profile analogous to the monoallelic behavior observed at the *IGF2*/*H19 *locus. Surprisingly, colocalization of CTCF and H3K9me3 does not provide a reliable measure of monoallelic binding of CTCF at the *IGF2BP1 *gene. Our studies included allele-specific sequencing of immunoprecipitated chromatin to demonstrate that chromatin at each *IGF2BP1 *allele is bivalent. Importantly, both alleles recruit RNA polymerase II, suggesting that silencing of one *IGF2BP1 *allele occurs after transcription initiation. By establishing which epigenetic configurations are involved in governing monoallelic gene expression, we will broaden the understanding of epigenetic mechanisms as they relate to cancer progression and cellular differentiation.

## Results

### Colocalization of CTCF and H3K9me3 in the human genome

Allele-specific binding of CTCF to the ICR regulates parent-of-origin-specific expression of the *IGF2 *gene and correlates with differential cytosine methylation and the presence of H3K9me3 [9-11]. We carried out a large-scale survey to identify genomic sites with chromatin markers similar to those at the ICR of the *IGF2*/*H19 *locus. Using ChIP-chip, we identified CTCF binding sites by tiling through the nonrepetitive portion of the genome in 100-bp intervals. Genomic sites bound by CTCF were assembled on a condensed array set that tiled through 9,823 sites using overlapping probes, and replicate ChIP experiments were performed. By using conservative criteria (positive signal in three replicates; *P *< 0.05) in this analysis, we identified 8,462 loci that interact with CTCF. To identify the subset of sites that associate with both CTCF and H3K9me3, we tested the association of these 8,462 loci with H3K9me3 using the condensed DNA array set. These analyses revealed 293 loci that are both bound by CTCF and marked by H3K9me3 (Table S1 in Additional file 1) (distances of CTCF and H3K9me3 peaks < 500 bp). Of the 293 loci, 115 directly mapped to coding regions. Of the remaining loci (174 of 293), the majority (147 loci) were located in intergenic regions at a distance > 10 kb to the nearest 5' end of known genes. Only 27 loci mapped to promoter regions. Overall, 40% of the CTCF/H3K9me3 loci mapped to intergenic regions, 51% mapped to intragenic domains and 9% mapped to promoter regions, a distribution similar to that of the 8,462 CTCF loci (44%, 51% and 10% respectively). Notably, the CTCF-regulated *IGF2*/*H19 *locus is included in the subset of 293 loci (Figure S1 in Additional file 2), suggesting that our experimental approach may be useful for the identification of similarly expressed genes.

### *IGF2BP1 *alleles are stochastically expressed in human B cells

Genes classified as "monoallelically expressed" encompass both imprinted genes, such as the *IGF2 *gene, where monoallelic expression is regulated in a parent-of-origin-specific manner, and stochastic loci, where individual alleles are randomly selected for expression independent of parental origin. In recent studies in which allele-specific transcription was assessed in several human cell lines, more than 300 (7.5%) of 4,000 human genes examined were subject to random monoallelic expression, with a majority of the latter being capable of biallelic expression [7].

To examine whether CTCF binding at sites marked by H3K9me3 is indicative of monoallelic expression, we first compared the genomic positions of our 293 loci with the list of genes expressed in a random allele-specific manner. Only a small number of genes (8 of 293 loci) were common to both the monoallelically expressed cohort described by Gimelbrant *et al*. [7] and our CTCF/H3K9me3 set of ChIP-chip binding loci.

**Figure 1:**
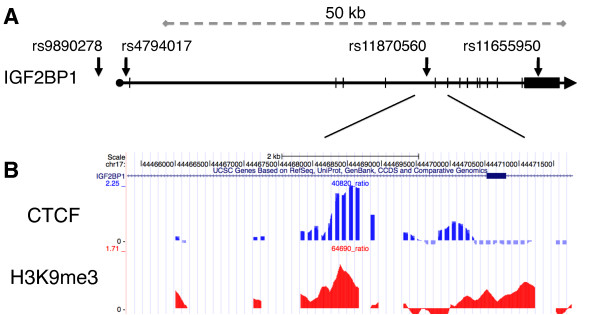
**Colocalization of CTCF and H3K9me3 at the *IGF2BP1 *locus**. Array-based chromatin immunoprecipitation (ChIP-chip) data for both CTCF and histone H3 trimethylated at lysine 9 (H3K9me3) identify candidate loci for analysis of monoallelic expression. **(A) **Depiction of the *IGF2BP1 *gene with specific SNPs examined in this study (arrows). **(B) **Close-up portion of the locus with tracks for CTCF enrichment (top track) and H3K9me3 association (bottom track) near SNP site rs11870560. The ChIP-chip data are displayed using the UCSC Genome Browser. DNA derived from CTCF ChIP experiments was analyzed by using microarrays with hybridization probes spaced 100 bp apart. The higher resolution of the H3K9me3 ChIP-chip data is due to the use of condensed array sets that tiled through all of the CTCF-positive regions with probes overlapping each other by 12 nt.

To further examine the correlation between CTCF/H3K9me3 and monoallelic expression, we selected 12 genes located near one of the 293 CTCF/H3K9me3 sites (*DIAPH1*, *FUS1*, *PKP1*, *ARFGAP2*, *PCDHGA*, *MTHFR*, *LAIR1*, *GPR3*, *ARMET*, *NPR1*, *NHLRC1 *and *IGF2BP1*) to search lymphoblastoid cell lines (LCLs) derived from a pedigree from the Center d'Etude du Polymorphisme Humaine (CEPH) for SNPs in exonic and 3'-UTR regions. The monoclonality of LCLs was confirmed by analysis of their immunoglobulin heavy chain (IgH) gene rearrangement (Figure S2 in Additional file 2) [22]. Sequencing of genomic DNA (gDNA) and cDNA of LCLs identified the insulin-like growth factor binding protein gene *IGF2BP1 *as the only candidate gene expressed from only one allele (Table S2 in Additional file 1). *IGF2BP1 *is an RNA-binding protein that regulates transcript stability and translation of the imprinted *IGF2 *gene [23]. In addition, *IGF2BP1 *binds to H19, MYC and β-TrCP1 mRNA to regulate message half-life, localization and translation of RNA, suggesting that the regulation of *IGF2BP1 *expression may affect disease and development [24,25]. We focused on *IGF2BP1 *to examine the contribution of CTCF and H3K9me3 markers colocalized at intron 5 to allele-specific expression (Figure 1).

**Figure 2:**
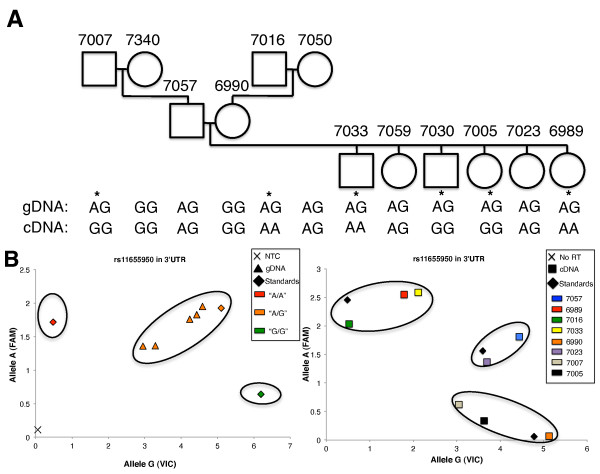
**Analysis of allele-specific *IGF2BP1 *expression**. Comparative analysis of sequence variations in B lymphoblasts of the CEPH pedigree family 1331 reveals monoallelic expression of the *IGF2BP1 *gene. **(A) **Pedigree analysis was carried out for the SNP site rs11655950 located in the 3'-UTR of the *IGF2BP1 *gene. Each individual is shown with CEPH family identification, sample identification and genetic information (SNP genomic DNA (gDNA) genotype- or transcript-derived genotype). Individuals with monoallelic *IGF2BP1 *gene expression are indicated by asterisks. If the individual is homozygous at the SNP, allele-specific expression cannot be defined. **(B) **Left: Genotyping results at rs11655950 with gDNA from members of CEPH family 1331. gDNA was analyzed using the TaqMan SNP Genotyping Assay. This assay discriminates between sequence variants using two allele-specific probes carrying two different fluorophores, VIC and FAM. Individuals coded in red and green represent cell lines that are homozygous for alleles A and G, respectively. Orange-labeled individuals contain both A and G alleles at SNP rs11655950 and represent informative cell lines used for further analysis of monoallelic expression. Diamonds indicate cDNA samples, and black × indicates averaged triplicates of a no-template control (NTC) near the origin of the graph. Right: Genotyping results of transcript-derived cDNA from heterozygous B lymphoblasts. Individuals are color-coded in the figure key. No-RT controls (No RT) from cDNA synthesis are shown near the origin of the graph and are indicated by a black X. Control samples (standards) of stem cell lines previously genotyped as homozygous AA, heterozygous AG and homozygous GG were plotted and are indicated by diamonds.

Sequencing of gDNA identified 10 individuals that were heterozygous at SNP rs11655950 in the 3'-UTR of *IGF2BP1 *(Figure 2A). All heterozygous SNPs were subsequently typed in cDNA. A comparison of the transcriptome-derived genotypes to genomic genotypes indicated that six individuals expressed *IGF2BP1 *primarily from only one allele. In contrast, four individuals were found to express both *IGF2BP1 *alleles (Figure 2A). SNP determination for genomic and cDNA for CEPH family 1331 was confirmed by allelic discrimination assays based on fluorogenic probes (TaqMan allelic discrimination assay; Applied Biosystems, Foster City, CA, USA), which yielded identical results (Figure S3 in Additional file 2). The TaqMan allelic discrimination assay, a real-time PCR based approach, yields a scatterplot of genotypes capable of quantitatively detecting a range of 1:1 and 1:5 ratios of individual alleles in DNA mixtures at SNP rs11655950 (Figure S4 in Additional file 2). Individuals GM7033 and GM6989 were found to express the paternally inherited *IGF2BP1 *allele, while GM7030 and GM7005 were found to express the maternally inherited allele (Figure 2). Individuals GM7007 and GM7016 also exhibited monoallelic expression of *IGF2BP1*, but we were unable to identify the mode of expression because of the limited pedigree. These data indicate that monoallelic expression at the *IGF2BP1 *gene locus is not determined by parent-of-origin markings; instead, it is defined by stochastic choice.

### CTCF binds to its target motif at the *IGF2BP1 *locus independently of DNA methylation

Binding of CTCF to its target motifs at both the human and mouse ICR of the *IGF2*/*H19 *locus is sensitive to DNA methylation [10,26]. To test whether monoallelic expression of *IGF2BP1 *in some individuals is also regulated by monoallelic DNA methylation of CTCF binding motifs, we examined a role for CpG methylation and allele-specific binding of CTCF at this locus.

To precisely determine the DNA sequence required for CTCF binding at the *IGF2BP1 *locus, we searched for potential motifs using SOMBRERO [27], a *de novo *motif-finding algorithm that uses multiple self-organizing maps (SOM) to cluster sequences of a specific length (reads) from a set of input sequences (such as enriched genomic loci identified by ChIP-chip experiments). Motif alignment using STAMP [28] and comparison to the JASPAR transcription factor database [29] identified a distinct cohort of 68 motif models, all of which were identical to the canonical CTCF motif previously reported (Figure S5 in Additional file 2) [30]. The clustered reads associated with all 68 motif models were mapped back to sequences enriched in our ChIP-chip analysis and were displayed using the UCSC Genome Browser (Figure S6 in Additional file 2). Using this approach, we identified 28,713 peaks, each composed of multiple overlapping reads, within the original 8,462 ChIP-chip loci. Using a strategy similar to that used to study ChIP-seq clustering [31], our frequency analysis of these peak heights yielded a bimodal distribution with an evident power law at low peak heights deviating to a clear excess in the numbers of peaks with heights > 10 (Figure S7 in Additional file 2). We consequently partitioned the peak populations into low-confidence and high-confidence groups using the peak height threshold of 10 (Figure S8 in Additional file 2).

**Figure 3:**
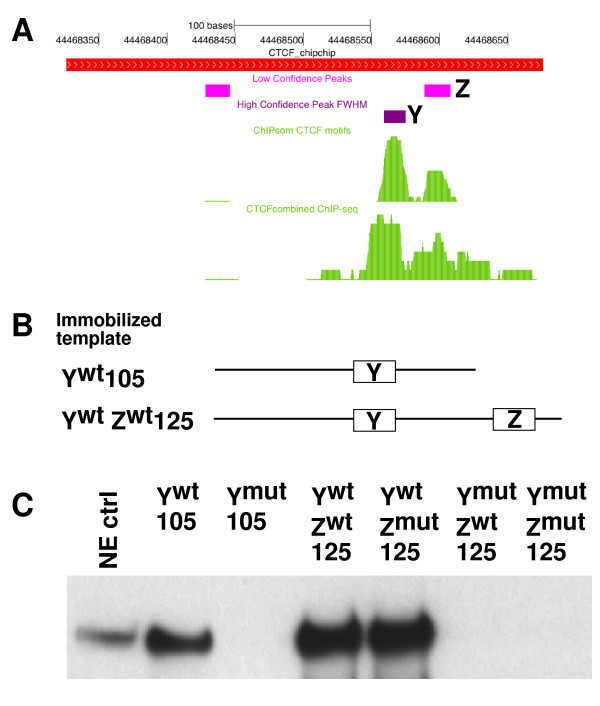
**Functional CTCF sequence motifs at the intronic region of the *IGF2BP1 *gene**. **(A) **UCSC Genome Browser display of relative positions of high- and low-confidence CTCF target motifs, ChIP-chip, ChIP sequencing (ChIP-seq) and ChIP self-organizing maps results. **(B) **Y^wt ^105-bp and Y^wt^Z^wt ^125-bp templates employed in the immobilized template assay. Detailed sequences of the templates are shown in Figure S9 in Additional file 2. **(C) **Western blot analysis of CTCF recruitment to Y^wt ^105-bp and Y^wt^Z^wt ^125-bp templates containing combinations of wild-type and mutated CTCF target sequences. Motif Y is sufficient for recruitment of CTCF.

Using this approach, we identified three potential motifs (X, Y and Z) (Figure 3) within the 350-bp region of the *IGF2BP1 *gene locus enriched in our ChIP-chip experiments. Two of the putative binding sites, Y and Z, accumulated a significant number of matches to motif models. However, only one of the three putative CTCF binding sites belongs to the group of high-confidence binding sites (site Y) (Figure 3). In support of our *in silico *analysis of CTCF binding, previously published high-resolution ChIP-seq data on CTCF binding revealed enrichment of sequences surrounding motifs Y and Z (Figure 3A), suggesting that either one or both motifs is required for CTCF recruitment.

To further define the contribution of motifs Y and Z to CTCF binding, we measured their ability to recruit CTCF *in vitro *using immobilized template assays (Figures 3B and 3C). Wild-type and mutant DNA templates containing either one or both motifs were linked to magnetic beads, incubated with nuclear extract, washed and tested for association with CTCF by performing Western blot analysis. A 105-bp template containing the wild-type *IGF2BP1 *intronic sequence efficiently recruited CTCF (Y^wt ^105-bp template) (Figure 3B). In contrast, CTCF binding was severely reduced when the putative CTCF motif Y was mutated by four base substitutions (Figure 3C and Figure S9 in Additional file 2). To test the contribution of the adjacent motif Z to CTCF binding at the *IGF2BP1 *locus, we generated several 125-bp DNA templates that encompassed both CTCF target motifs (Figure 3B). Targeted mutations at specific positions of motif Y and/or motif Z were introduced to test the contribution of each motif to recruitment of CTCF. Detailed sequences are shown in Figure S9 in Additional file 2. As shown in Figure 3C, the 125-bp template recruited CTCF more efficiently than the 105-bp template. However, motif Z does not contribute to CTCF recruitment, since targeted mutations in motif Z do not influence the level of CTCF binding. Consistent with this notion, CTCF binding is undetectable in the absence of a wild-type motif Y (Figure 3C).

**Figure 4:**
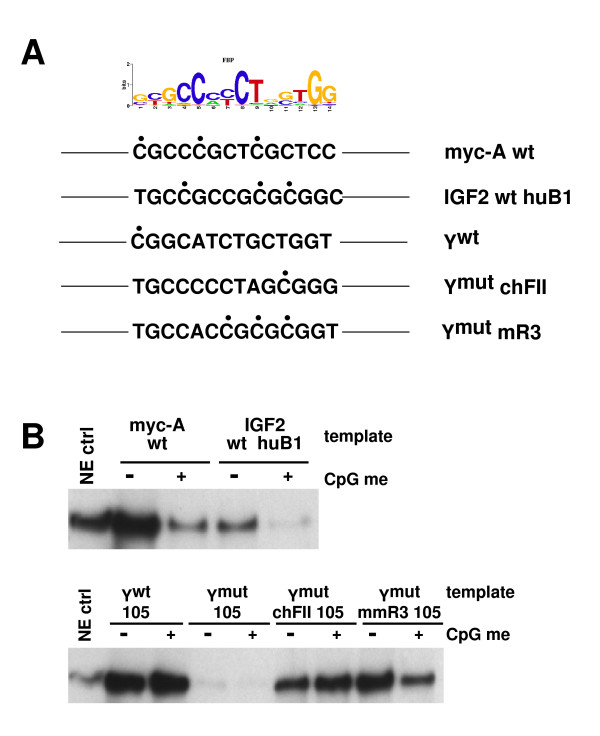
**Cytosine methylation of the CTCF core motif Y does not influence binding of CTCF**. **(A) **CTCF motifs used in the context of the 105-bp immobilized template derived from the intronic region of the *IGF2BP1 *gene are shown. The position frequency matrix of the CTCF target motif is shown at the top. Only the sense strand of the motifs is shown. CpG residues are indicated by filled black circles. Myc-A, IGF2 huB1 and Y^wt ^are CTCF target sequences derived from *MYC*, *IGF2 *and *IGF2BP1 *gene loci. Y^mut ^chFII and Y^mut ^mmR3 contain the CTCF target sequence of the chicken HS4 insulator [57] and the CTCF target region of the mouse imprinting control region R3 [10]. **(B) **Top: control experiments revealed the sensitivity of CTCF binding to DNA methylation (CpG me) at the myc-A and IGF2 huB1 templates. Bottom: methylation of the 105-bp Y^wt ^template did not affect the recruitment of CTCF. While methylated chicken FII CTCF target sites efficiently recruited CTCF, CpG methylation of the mouse R3 sequence decreased the binding of CTCF.

CTCF binding site Y at the *IGF2BP1 *gene contains a single CpG residue adjacent to the 14-bp core sequence of CTCF (Figure 4A). To establish whether binding of CTCF to Y^wt ^is inhibited by cytosine methylation, we tested Y^wt ^105-bp immobilized templates after *in vitro *methylation of cytosine residues by CpG methyltransferase M.Sssl. For comparison, we examined CTCF motifs containing a higher CpG content, including site A of the MYC gene [32] as well as the B1 sequence of the ICR of the human *IGF2*/*H19 *locus [10]. Cytosine methylation at the human B1 sequence is known to inhibit binding of CTCF. Consistent with this, recruitment of CTCF *in vitro *to immobilized templates containing the B1 sequence or the MYC site A is highly sensitive to DNA methylation (Figure 4B, top). In contrast, CpG methylation of the Y^wt ^motif has no effect on CTCF recruitment. Replacement of the Y^wt ^core motif by the CTCF-binding sites of the chicken FII insulator element yields similar results. However, CTCF binding becomes sensitive to CpG methylation upon modification of the core motif to the mouse R3 sequence, a homologue of the human B1 sequence. In combination, despite the presence of a methylable CpG residue, binding of CTCF to the Y^wt ^sequence of the *IGF2BP1 *gene *in vitro *is not sensitive to CpG methylation.

**Figure 5:**
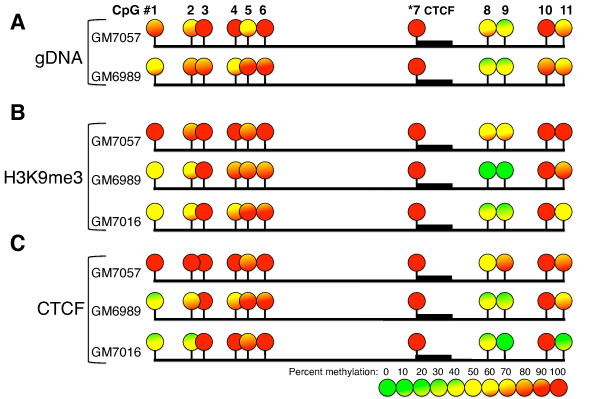
**DNA methylation analysis of the *IGF2BP1 *CTCF binding region**. Analysis of DNA methylation with bisulfite sequencing at the intronic CTCF binding region of the *IGF2BP1 *gene is shown. **(A) **The percentage of methylation of CpG sites in gDNA derived from cell lines that express *IGF2BP1 *from only one allele (GM7016, GM6989) or from both alleles (GM7057) is shown. The CpG residue located within the CTCF binding motif is invariably methylated and is indicated by the thick black bar located adjacent to CpG site 7 (indicated by asterisks). **(B) **The percentage of methylation at each CpG site of the *IGF2BP1 *CTCF site in DNA samples recovered from anti-H3K9me3 ChIP. **(C) **The percentage of methylation at each CpG site of the *IGF2BP1 *CTCF site in DNA samples recovered from anti-CTCF ChIP experiments. The level of DNA methylation is represented according to the heat map keys located at the bottom of the figure.

To confirm that our *in vitro *characterization of CTCF binding accurately reflected the *in vivo *association of CTCF with the *IGF2BP1 *locus, we evaluated the methylation status of the CTCF motif and adjacent CpG residues in the *IGF2BP1 *intronic region in both biallelically (GM7057) and monoallelically (GM6989) expressing cells by using bisulfite sequencing (Figure 5). The methylation levels were calculated using BiQ Analyzer software [33]. Our data reveal that the CpG residue at the 5' end of the CTCF binding motif Y is invariably methylated. In addition, other methylable residues in this region exhibited some degree of DNA methylation. To further confirm binding of CTCF to methylated *IGF2BP1 *intronic sequences, we bisulfite-sequenced DNA derived from immunoprecipitates of ChIP experiments with CTCF antibodies. As a control, we bisulfite-sequenced the *IGF2BP1 *region derived from anti-H3K9me3 ChIP experiments. The results confirmed our *in vitro *finding that demonstrated an association of CTCF with a methylated motif (Figures 5B and 5C).

### CTCF and H3K9me3 colocalize at both the maternal and paternal *IGF2BP1 *alleles

**Figure 6:**
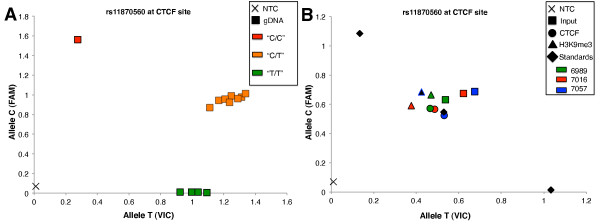
**Allelic specificity of CTCF and H3K9me3**. Informative ChIP templates were analyzed using the TaqMan allelic discrimination assay to address the allelic association of CTCF and H3K9me3. **(A) **Genotyping results at rs11870560 identify informative cell lines useful for the detection of allele-specific association of CTCF and H3K9me3. gDNA obtained from monoallelic and biallelic cell lines were genotyped using the TaqMan allelic discrimination assay. Squares represent gDNA samples and are coded in red and green to represent cell lines that are homozygous for allele C and allele T, respectively. Orange indicates heterozygous individuals. Averaged triplicate of a no-template control (NTC) is shown near the origin of the graph. **(B) **Genotyping at SNP rs11870560 with DNA templates recovered from ChIP experiments was used to identify the enrichment of the two alleles with either CTCF (circle) or H3K9me3 (triangle). Each color shown in the figure key represents a lymphoblastoid cell line (LCL) derived from an individual of the pedigree, while the shape represents the source of each sample (for example, squares signify input samples, while circles and triangles indicate ChIP samples obtained with CTCF and H3K9me3 antibodies, respectively). Immunoprecipitated templates were generated using the ChIP protocol described in Materials and Methods. Both monoallelic and biallelic cell lines indicate biallelic distribution of both CTCF and H3K9me3. Diamonds indicate control LCL samples (standards) previously genotyped as homozygous CC, heterozygous CT and homozygous TT.

Consistent methylation of the CTCF-binding motif in *IGF2BP1 *indicated that DNA methylation is not allele-specific. To directly determine whether CTCF is bound monoallelically, we determined the allele-specific association of both CTCF and H3K9me3 by sequencing DNA recovered from ChIP experiments. We first identified informative cell lines by genotyping individuals from CEPH pedigree 1331 at SNP sites located close to the CTCF binding site. Cell lines derived from both monoallelically (GM7016 and GM6989) and biallelically (GM7057) expressing individuals were heterozygous at SNP site rs11870560 at the CTCF site (Figure 6A). We first applied the allelic discrimination assay to serial dilutions of known homozygotes of the two possible alleles to test its ability to quantitatively assess the contribution of each allele in a DNA mixture. This assay provides quantitative results with high sensitivity and reproducibility within a ten-fold range of DNA concentrations, thus making it a useful tool for allelic discrimination of immunoprecipitated DNA (Figure S4 in Additional file 2). We used two monoallelically (GM7016 and GM6989) and one biallelically (GM7057) expressing cell lines to genotype DNA recovered from ChIP assays using either anti-CTCF or anti-H3K9me3 antibodies. Each analysis was performed in triplicate. Equal proportions of the two sequence variants were detected in DNA derived from ChIP assays with either H3K9me3 or CTCF antibodies, indicating that CTCF associates with both the maternal and paternal alleles (Figure 6B). Thus, monoallelic expression of the *IGF2BP1 *gene is not mediated through monoallelic binding of CTCF.

### The *IGF2BP1 *promoter associates with both active and silent histone modifications in B cells

**Figure 7:**
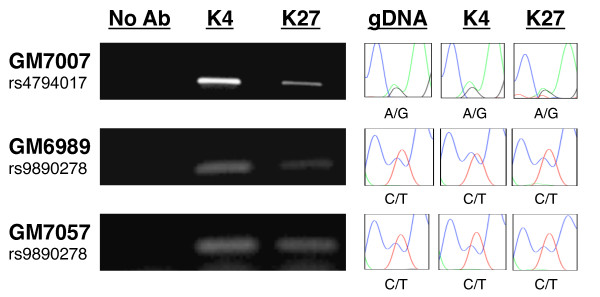
***IGF2BP1 *promoter region is enriched with activating and silencing chromatin modifications**. DNA recovered from ChIP experiments using anti-H3K4me3, anti-H3K27me3 and RNA polymerase II ChIP templates was genotyped by sequencing the *IGF2BP1 *promoter region containing sequence variant rs4794017 or rs9890278. Left: Enrichment of H3K4me3 (K4) and H3K27me3 (K27) in monoallelically (GM7007, GM6989), and biallelically (GM7057) expressing cell lines. The positions of informative SNPs rs479017 and rs9890278 are shown in Figure 1. Both activating and silencing marks are significantly enriched. Right: Sequences enriched by ChIP were excised and sequenced. The results show an association of both alleles with active and silent histone modifications at the *IGF2BP1 *promoter region independent of transcriptional status.

To define alternative mechanisms responsible for random monoallelic expression of *IGF2BP1*, we sought to identify markers that distinguish the active and inactive alleles. K27-trimethylated and K4-trimethylated histone H3, respectively, mark transcriptionally silent and active chromatin. We determined the relative enrichment of these two histone markers at the *IGF2BP1 *promoter for each allele in both monoallelically and biallelically expressing cell lines using ChIP with anti-H3K4me3 and anti-H3K27me3 antibodies. Both H3K4me3 and H3K27me3 were detected at the *IGF2BP1 *gene promoter (Figure 7A). To determine whether any of the histone modifications selectively associates with either allele, we again searched for informative sequence SNPs at the *IGF2BP1 *promoter region in the CEPH pedigree. Cell lines derived from individuals GM6989 (monoallelically expressing cell line) and 7057 (biallelically expressing cell line) were heterozygous at SNP rs9890278 located upstream of the transcription initiation site, whereas GM7007 (monoallelically expressing cell line) was heterozygous for SNP rs4794017 located 1 kb downstream of the transcription initiation site. To address whether active and silent alleles in these cell lines are distinguished by specific histone markers, we sequenced SNPs rs9890278 and rs4794017 in gDNA recovered from ChIP experiments using anti-H3K4me3 and anti-H3K27me3 antibodies. The results revealed that both H3K4me3 and H3K27me3 are detected on both alleles in a bivalent fashion (Figure 7). In combination, our results indicate that both active and silent histone markers (H3K4me3 and H3K27me3) coexist in the promoter region of both *IGF2BP1 *alleles in monoallelically as well as biallelically expressing cell lines. These data indicate that allele-specific expression of *IGF2BP1 *cannot be explained by differential association of active and silent histone markers.

### Silencing of the inactive *IGF2BP1 *allele by inhibition of RNA polymerase II elongation

**Figure 8:**
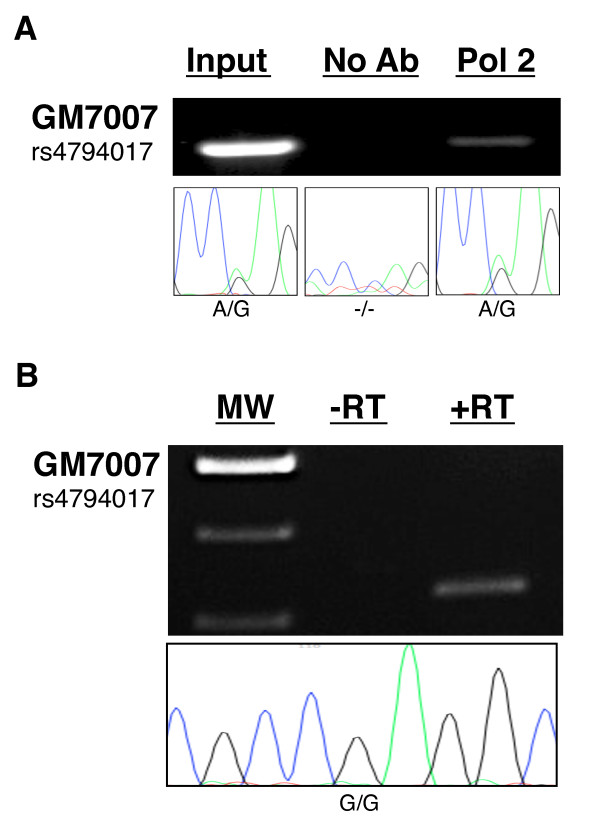
**RNA polymerase II associates with both alleles in a monoallelically expressing cell line**. **(A) **Recruitment of RNA polymerase II to the *IGF2BP1 *promoter was examined by ChIP in monoallelically expressing GM7007 cells. DNA recovered from chromatin that had been immunoprecipitated with anti-RNA polymerase II antibodies (Pol2) was amplified and sequenced for allelic association. Sequencing results (bottom) reveal that both alleles of the monoallelically expressing cell line GM7007 associate with RNA polymerase II near SNP site rs4794017. In contrast, sequencing of DNA from "no antibody" ChIP reactions failed to produce sequence reads. **(B) **Allele specificity of precursor mRNA was determined by sequencing of cDNA prepared from total RNA of GM7007 cells. RNA had been extensively pretreated with DNase I to eliminate gDNA prior to reverse transcription by RT. Subsequently, cDNA samples were amplified using primers flanking rs4794017. In the absence of RT (-RT), no amplification products were oberved. +RT amplicons were gel-purified and sequenced. Bottom: Sequence traces at the heterozygous SNP site rs4794017 located 1 kb downstream of the transcription initiation site in cDNA of GM7007 indicate a single allele.

Monoallelic expression of *IGF2BP1 *cannot be attributed solely to selective activation or silencing of one allele through histone modifications, since H3K4me3 as well as H3K27me3 are detected at both alleles. H3K4me3 is typically associated with transcriptionally active alleles, raising the question whether allele-specific transcription elongation or RNA processing accounts for monoallelic expression of the *IGF2BP1 *gene. To address this hypothesis, we again searched mono- and biallelically expressing cell lines for sequence SNPs near the site of transcription initiation at the *IGF2BP1 *promoter. Within CEPH pedigree 1331, only line GM7007 contained a heterozygous genotype at SNP site rs4794017 located within intron 1, 1 kb downstream of the transcription initiation site. We performed RNA polymerase II ChIP on chromatin prepared from this monoallelically expressing line. Quantitative real-time PCR analyses revealed enrichment of *IGF2BP1 *promoter sequences similar to the enrichment observed at the MYC promoter. Immunoprecipitated DNA was PCR-amplified and sequenced (Figure 8A). Identification of both sequence variants at rs4794017 in DNA recovered from ChIP experiments indicates that RNA polymerase II is associated with both *IGF2BP1 *alleles, which is consistent with the presence of H3K4me3 at the promoter of both alleles.

These data suggest that allele specificity of transcription is achieved after recruitment of RNA polymerase to both alleles, such as through transcriptional pausing and/or selective RNA processing. A major rate-limiting step in transcription elongation is pausing of RNA polymerase II in the promoter proximal region immediately downstream of the transcription initiation site [34-37]. We sequenced the 5' portion of the *IGF2BP1 *gene of all monoallelically expressing cell lines to identify sequence variants that would be useful for allelic identification of promoter proximal regions occupied by RNA polymerase II or for the determination of the allelic origin of unspliced, precursor pre-mRNA transcripts. Since no additional informative sequence variants were identified, we focused on the detection and sequencing of pre-mRNA transcripts about 1 kb downstream of the transcription initiation site in GM7007. Using the informative SNPs located within intron 1 of this gene, we targeted nascent unspliced RNA with primers designed to amplify a region containing SNP site rs4794017. To avoid detection of gDNA in RNA samples, DNA was efficiently removed by treatment with an engineered, highly active form of DNase I (TURBO DNase I; Applied Biosystems/Ambion, Austin, TX, USA). This protocol allowed detection of pre-mRNA free of gDNA contamination (Figure 8B). Sequencing of amplified *IGF2BP1 *pre-cDNA revealed only one of the two sequence variants at SNP rs4794017, indicating that pre-mRNA transcripts are transcribed from only one allele despite the presence of RNA polymerase II on both alleles. Thus, our data indicate that monoallelic expression of the *IGF2BP1 *gene is regulated through allele-specific transcriptional elongation prior to SNP site rs4794017, located approximately 600 bp downstream of the first intron splice site.

## Discussion

Allele-specific expression in which one parental allele is stochastically or parent-of-origin-specifically silenced is widespread in mammalian organisms. Large-scale, allele-specific gene expression analyses have revealed that 5% to 10% of autosomal genes show random monoallelic transcription [7]. The stability of allele-specific expression through many cell passages suggests that epigenetic modifications maintain this specific type of gene regulation throughout generations of cells. Analogously to the regulation at the imprinted *IGF2*/*H19 *locus, we tested the hypothesis whether monoallelic binding of CTCF, a characteristic marker for the *IGF2*/*H19 *ICR, also underlies random monoallelic expression. Using ChIP-chip analyses, we identified chromosomal loci that are enriched in both CTCF and H3K9me3 and cross-correlated their positions with previously published lists of monoallelically expressed genes. Our data indicate that genomic loci enriched for both CTCF and H3K9me3 do not significantly correlate with monoallelically expressed genes. While this lack of correlation could be formally attributed to variations in monoallelic expression between different cell lines and types, it should be noted that the genome-wide pattern of CTCF binding is very consistent between different cell lineages [30,38,39]. Thus, if CTCF and H3K9me3 contribute to allele-specific expression, it should be detectable through allele-specific association of CTCF and H3K9me3. Focusing on the *IGF2BP1 *gene, we tested whether monoallelic expression in a pedigree of LCLs correlates with monoallelic binding of CTCF. Although binding of CTCF to its targets is thought to be sensitive to DNA methylation, we surprisingly found the cytosine residue closely flanking the CTCF target motif at the *IGF2BP1 *gene to be consistently methylated without any effect on CTCF recruitment. Indeed, our *in vitro *analyses of the binding requirements using immobilized templates confirmed that methylation of cytosine residues within the *IGF2BP1 *sequence does not affect CTCF binding. These data are consistent with those in previous studies in which researchers found that cytosine methylation outside the CTCF core motif did not affect the binding affinity of bacterially expressed wild-type and mutant CTCF proteins [40]. This information is useful for the identification of the genomic subset of CTCF sites that might contribute to differential cell- and stage-specific expression due to their sensitivity to cytosine methylation, potentially mediating changes in large-scale chromatin organization during development and disease.

A number of studies have examined the correlation of allele-specific expression with allele-specific association of epigenetic markers [21,41-45]. The data produced by these studies have established common signatures of imprinted alleles, including H3K9me3 and H3K4me3, providing a powerful means by which to identify novel imprinted or monoallelically expressed loci [46-48]. In contrast to the strict allele-specific association of DNA methylation and chromatin markers at imprinted genes, histone modifications at the nonimprinted, monoallelically expressed *IGF2BP1 *gene do not predict the active allele. Both H3K4me3 and H3K27me3, markers characteristic of active and inactive loci, are associated with each allele, as both sequence variants of SNP rs4794017 are present in the DNA of heterozygous individuals recovered from ChIP experiments. Moreover, loading of RNA polymerase II also does not provide a reliable marker for identifying the transcribed allele. Our ChIP experiments identified both sequence variants at SNP rs4794017 within the promoter proximal region of anti-RNA polymerase II immunoprecipitated DNA. Because only one LCL in our study was informative for determining an association of RNA polymerase II at the *IGF2BP1 *alleles, we could not define how frequently this type of regulation occurs within cell lineages and throughout the genome. However, other investigators have reported similar results at the *PCNA *gene. Maynard *et al*. [44] found that both *PCNA *alleles in IMR90 cells are bound by RNA polymerase II, although only one allele generates full-length mRNA. Together, these data suggest that transcription elongation not only is a general rate-limiting step in the transcription of the vast majority of genes [34,35,37] but also regulates the expression of a subset of monoallelically expressed genes.

The expression of *IGF2BP1 *in differentiated cell types, including LCLs, is significantly lower than in embryonic stem cells. In an attempt to determine whether allele-specific expression also contributes to *IGF2BP1 *regulation early in development, we genotyped both gDNA and cDNA in 11 human embryonic stem cell (hESC) lines. However, while only three hESC lines were informative (heterozygous at SNP rs11655950), all three expressed *IGF2BP1 *in a biallelic manner. Although the number of available and informative hESC lines is not sufficient to clearly define a role for allele-specific elongation in early developmental stages, we believe that it is unlikely that this mechanism is restricted to cell types with low levels of *IGF2BP1 *expression. Control of transcriptional activity through promoter proximal pausing or premature termination of transcription is not restricted to specific gene classes characterized by low levels of transcriptional activity [35]. We speculate that distinct positioning of the homologous alleles within the nuclear space and association with distinct "transcription factories" may contribute to monoallelic transcription elongation.

The *IGF2BP1 *gene is highly expressed during embryonic development and is required for the regulation of mRNA stability of several genes involved in growth regulation, including the *IGF2*, β-catenin and *MYC *genes [23-25]. Consistent with its role in early developmental stages, the *IGF2BP1 *gene is downregulated in differentiated cell types, and overexpression of *IGF2BP1 *is known to occur in multiple human cancers, including breast, lung and colon [49-52]. Thus, changes in the level of *IGF2BP1 *expression through silencing of only one allele could provide a safeguard against pathogenesis and disease.

## Conclusions

Allele-specific gene expression is common in the human genome and is thought to contribute to phenotypic variation. The allele-specific association of CTCF, H3K9me3 and DNA methylation is a characteristic marker of imprinted gene expression at the *IGF2*/*H19 *locus, raising the question whether these epigenetic markers are useful for identifying both imprinted and random monoallelically expressed genes throughout the genome. In this study, we have demonstrated that colocalization of CTCF and H3K9me3 does not represent a reliable chromatin signature indicative of monoallelic expression. In addition, we conclude that allele-specific binding of CTCF requires methylation of very specific cytosine residues within the target motif, effectively limiting the number of CTCF binding sites potentially affected by allele-specific binding. In addition, the active and inactive alleles of random monoallelically expressed genes do not necessarily correlate with active or inactive histone markers. Remarkably, the selection of individual alleles for expression at the *IGF2BP1 *locus occurs during early stages of transcription elongation.

## Methods

### ChIP-chip analyses

The amplification and preparation of immunoprecipitated DNA derived from HBL100 cells for hybridization to ENCODE arrays (Roche NimbleGen Inc., Madison, WI, USA) was performed essentially as described previously [53]. Sample labeling and array hybridization were performed at NimbleGen Systems Inc. Genomic control DNA was labeled with Cy3, and sample DNA was labeled with Cy5. Both Cy3- and Cy5-labeled DNA were hybridized to high-density arrays tiling through ENCODE regions with 50-mer oligonucleotides across nonrepetitive genomic regions. The ratios of the Cy3 and Cy5 intensities of each probe were calculated using NimbleGen Systems' proprietary software.

### Peak detection and false-positive rate calculation

A genomic sequence was considered a possible CTCF-binding site if there were at least four probes among the sequence probe and the flanking probes within a window covering 250 bp on both sides of the probe had log_2 _ratio values above a specified cutoff value. The cutoff value was calculated separately for each chromosome. The cutoff value is a given percentage of the value (mean + 6 × standard deviation) of the log_2 _ratio values of all the probes covering the chromosome. The possible binding sites thus detected are called peaks. To calculate the false-positive rate (FPR) by data permutation, the log_2 _ratio values among probes were scrambled to generate a randomized data set for each individual chromosome. Multiple repetitions of this process generated 20 randomized data sets for each chromosome. Subsequently, the peak detection algorithm described above was applied to count the average number of peaks in the 20 randomized data sets using the same cutoff. The ratio of that number to the number of peaks from the nonrandomized data set is the FPR. The FPR is associated with the threshold setting, which is indicated by the value of cutoff *P*. Peak detection and randomization of data sets were repeated for different threshold settings of *P*. The corresponding FPRs were calculated and assigned to peaks. The FPR value assigned to the individual peaks is the value associated with the cutoff *P *at which the peak is first detected.

Peak discovery was performed using chromatin immunoprecipitate:input ratios combined from adjacent oligonucleotides within 250-bp regions. The FPR of detection was estimated by permutation analyses in which the experimentally determined log_2 _ratio values were reassigned to probes in a random fashion, allowing selection of stringency and specificity levels. To define sites of CTCF interaction with high confidence, peaks were required to be present in all three biological replicates and to be generated at a FPR < 0.05.

### Chromatin immunoprecipitation

Chromatin was prepared for immunoprecipitation as described previously [54] by cross-linking the cells in 1% formaldehyde for 5 minutes and subjecting them to subsequent sonication until the bulk of DNA was 300 to 600 bp in size. Chromatin corresponding to 2 × 10^7 ^cells was immunoprecipitated with anti-CTCF antibody (D31H2; Cell Signaling Technology, Danvers, MA, USA), anti-H3K9me3 antibody (ab8898; Abcam, Cambridge, MA, USA), anti-trimethyl K4-histone H3 antibody (ab8580; Abcam), anti-trimethyl K27-histone H3 antibody (Millipore 07-449, Billerica MA, USA) or anti-RNA polymerase II antibody (sc899; Santa Cruz Biotechnology, Santa Cruz, CA, USA). Immunoprecipitates were washed, the DNA protein cross-links were reversed and the recovered DNA was tested by performing conventional quantitative PCR as described previously [54]. RNA polymerase II ChIP experiments were performed using the Matrix ChIP protocol [55]. Sequences of primers specific for the gene loci under study as well as the reference primers are available upon request.

### RNA extraction and RT-PCR

Synthesis of cDNA was carried out according to the manufacturer's instructions (Qiagen, Valencia, CA, USA) using 1 μg of total RNA. For detection of pre-mRNA, RNA preparations were pretreated with TURBO DNase I (Ambion/Applied Biosystems) as described in the manufacturer's protocol. RT was carried out at 37°C for one hour.

### Cell culture

Cell lines were cultured in RPMI 1640 medium supplemented with 10% FCS, 2 mM L-glutamine and the antibiotics penicillin (50 U/mL) and streptomycin.

### Sodium bisulfite conversions

gDNA was treated with sodium bisulfite using the EZ DNA Methylation Kit (Zymo Research, Orange, CA, USA) according to the manufacturer's instructions. PCR amplification of bisulfite-treated DNA was performed using ZymoTaq DNA Polymerase (Zymo Research Corporation, Irvine, CA, USA) and conversion-specific primers targeted to the *IGF2BP1 *CTCF region (forward primer: 5'-TATTTTTTAGTTGGGTTAAT-TGGTG-3', reverse primer: 5'-ATACTACCTCTCCTTCCAAAATCTC-3'). The amplified products were purified by gel electrophoresis and sequenced. Each case was scored as methylated or unmethylated, and the percentage of methylation was calculated using BiQ Analyzer software [33].

### TaqMan allelic discrimination assays

TaqMan allelic discrimination assays were performed according to the manufacturer's instructions with the following adjustments: cDNA from B lymphoblasts was preamplified for 14 cycles. PCR products were gel-purified and subsequently used as templates in the genotyping of samples. The specific primer sequences used are avaliable upon request.

### *In vitro *CTCF binding analysis using immobilized templates

Crude nuclear extract was prepared from 1 × 10^9 ^Jurkat cells grown in growth media (RPMI 1640 with 10% fetal bovine serum) according to methods described previously [56]. Biotinylated template DNA was generated by PCR amplification of the *IGF2BP1 *intronic region using a biotinylated/nonbiotinylated primer combination. The specific primer sequences are available upon request. For each binding reaction, 1 pM biotinylated DNA template was coupled to 50-μg streptavidin-linked magnetic beads (Dynabeads M-280 Streptavidin; Invitrogen, Carlsbad, CA, USA). Templates immobilized to magnetic beads were washed three times in B&W buffer (5 mM Tris, pH 7.5, 0.5 mM ethylenediaminetetraacetic acid (EDTA), 1 M NaCl) and resuspended in Jurkat nuclear extract. After a two-hour incubation at 4°C, immobilized templates were washed three times in Dignam buffer D (20 mM 4-(2-hydroxyethyl)-1-piperazineethanesulfonic acid, pH 7.9, 20% glycerol, 0.1 M KCl, 1 mM EDTA, 0.1 mM ethylene glycol tetraacetic acid, 1% Nonidet P-40, 1 mM dithiothreitol) containing protease inhibitor (P8340; Sigma, St Louis, MO, USA). To recover template-bound proteins, beads were incubated in elution buffer (5 mM Tris, pH 7.5, 0.5 mM EDTA, 1 M NaHCO_3_) including protease inhibitors. After a 5-minute incubation, the eluate was removed and transferred into a fresh tube. The presence of CTCF in the eluate was determined using standard Western blot analysis protocols.

## Abbreviations

FCS: fetal calf serum; PCR: polymerase chain reaction; RT: reverse transcriptase; SNP: single-nucleotide polymorphism.

## Competing interests

The authors declare that they have no competing interests.

## Authors' contributions

AK conceived of and designed the study. BJT, EDR and AK performed the experiments. PÓB, AAG, JMG and NK provided bioinformatics support and carried out the statistical analyses. PW and KB contributed the samples. BJT, PW, AAG and AK drafted the paper. All authors read and approved the final manuscript.

## Supplementary Material

Additional file 1 **Table S1. Genomic coordinates of 293 genomic sites that are marked by both CTCF and H2K9me3**. Table S2. List of genes tested for monoallelic expression in lymphoblastoid cell lines. Click here for file

Additional file 2 **Figure S1. Detection and colocalization of CTCF and H3K9me3 at the human IGF2-H19 ICR locus by ChIP-chip experiments**. Top: Enrichment of CTCF binding sites. Middle: Results of large-scale array-based chromatin immunoprecipitation (ChIP-chip) survey of histone H3 trimethylated at lysine 9 (H3K9me3) binding. Bottom: H19 exons demonstrating positions of CTCF binding and histone modifications relative to exons. **Figure S2. Analysis of the clonal status of lymphoblastoid cell lines used in this study**. Following the protocol described in [22], PCR amplification of two regions within the variable segment in the immunoglobulin heavy chain gene (conserved framework region 2 (Fr2) and the variable joining regions (VLJH)) reveals the clonal status of lymphoblastoid cell lines (LCLs). The amplification product from a polyclonal population (P) gives rise to fragments of varying length due to the large number of rearranged immunoglobulin genes and appears as a broad band. Amplification of DNA derived from monoclonal cell lines results in one or two discrete bands within an expected size range of 240 to 280 bp. The polyclonal sample (P) was obtained from the peripheral blood of a healthy donor. Lanes 1 through 4: monoclonal cell lines GM7007, GM7033, GM6989 and GM7030. Lanes 5 through 8: monoclonal lines GM7050, GM7023, GM7059 and GM7057. MW, DNA size marker. **Figure S3. Sequencing results give results identical to those derived from the TaqMan allelic discrimination assay**. **(A) **Standard sequencing results of two individuals at SNP site rs9904288. **(B) **TaqMan allelic discrimination assay confirms the heterozygosity of GM7057 and the homozygosity of GM6990. **Figure S4. Quantitative assessment of TaqMan genotyping using specific probe set at SNP rs11655950**. The 3'-UTR of the *IGF2BP1 *gene was amplified using primers given in Supplemental Table 2. This segment contains an A/G SNP. The PCRs included a FAM-labeled probe for the A allele and a VIC-labeled probe for the B allele. After PCR amplification, an end point fluorescence reading was taken on the ABI PRISM 7700 with SDS version 1.4 software (Applied Biosystems). The determination of the quantitative assignment of known genotypes is plotted. Concentration dilutions were created using known homozygous cell lines. Preparations of gDNA samples shown represent the following allele B/allele A ratios: 100:0, 80:20, 60:40, 50:50, 40:20, 20:80 and 0:100. Heterozygosity was based on the fluorescence intensity of FAM, VIC or both dyes together. Error bars indicate 5% of triplicate sample value. Allele A curve yields *y *= 0.0102*x *+ 0.0415 with *R*^2 ^= 0.98934. Allele B curve yields *y *= -0.0085*x *+ 0.9796 with *R*^2 ^= 0.98196. **Figure S5. Phylogenetic tree of motifs determined from motif analysis of the 8,462 loci derived from the ChIP-chip analysis using STAMP**. All members of the highlighted group have matches identical to the canonical CTCF motif model as part of the JASPAR transcription factor binding site database. The resulting familial binding profile for all 68 such models is displayed. **Figure S6. Fine mapping of CTCF motifs in sequences enriched in ChIP-chip experiments**. Motif reads were mapped onto the genomic loci defined by ChIP-chip for CTCF binding. The extent of the ChIP-enriched sequences is indicated by red bar. Several read clusters are apparent and vary in depth and spatial extent (green areas). **Figure S7. Frequency distribution of cluster depth for all motif clusters**. A power law is apparent for clusters of depth ≤ 10 with evident deviation in the population and a maximum of about 40. The vertical green line demarcates the low and high confidence clusters. **Figure S8. Discrimination between high- and low-confidence sites**. The region shown in Supplemental Figure S6 is annotated by overlaying enriched sequences with high- and low-confidence tracks. **Figure S9. Sequences of immobilized templates used in *in vitro *binding experiments**. CTCF core motifs Y and Z are underlined. Site-specific mutations in either the Y or Z motif are highlighted in yellow. In Y^mut ^chFII and Y^mut ^mmR3, site-specific mutations (highlighted in green) were introduced to generate CTCF motifs identical to the chicken HS4 FII site and the mouse imprinting control region R3. The *IGF2 *wild-type huB1 sequence is derived from the human *IGF2 *imprinting control region containing the methylation-sensitive CTCF binding site B1. Click here for file

## References

[B1] Delaval K Feil R Epigenetic regulation of mammalian genomic imprinting Curr Opin Genet Dev 2004 14 188 195 10.1016/j.gde.2004.01.005 15196466

[B2] Ferguson-Smith AC Surani MA Imprinting and the epigenetic asymmetry between parental genomes Science 2001 293 1086 1089 10.1126/science.1064020 11498578

[B3] Gregg C Zhang J Weissbourd B Luo S Schroth GP Haig D Dulac C High-resolution analysis of parent-of-origin allelic expression in the mouse brain Science 2010 329 643 648 10.1126/science.1190830 20616232 PMC3005244

[B4] Chess A Simon I Cedar H Axel R Allelic inactivation regulates olfactory receptor gene expression Cell 1994 78 823 834 10.1016/S0092-8674(94)90562-2 8087849

[B5] Bix M Locksley RM Independent and epigenetic regulation of the interleukin-4 alleles in CD4^+ ^T cells Science 1998 281 1352 1354 9721100 10.1126/science.281.5381.1352

[B6] Holländer GA Zuklys S Morel C Mizoguchi E Mobisson K Simpson S Terhorst C Wishart W Golan DE Bhan AK Burakoff SJ Monoallelic expression of the interleukin-2 locus Science 1998 279 2118 2121 10.1126/science.279.5359.2118 9516115

[B7] Gimelbrant A Hutchinson JN Thompson BR Chess A Widespread monoallelic expression on human autosomes Science 2007 318 1136 1140 10.1126/science.1148910 18006746

[B8] Reik W Walter J Genomic imprinting: parental influence on the genome Nat Rev Genet 2001 2 21 32 11253064 10.1038/35047554

[B9] Bell AC Felsenfeld G Methylation of a CTCF-dependent boundary controls imprinted expression of the *Igf2 *gene Nature 2000 405 482 485 10.1038/35013100 10839546

[B10] Hark AT Schoenherr CJ Katz DJ Ingram RS Levorse JM Tilghman SM CTCF mediates methylation-sensitive enhancer-blocking activity at the *H19*/*Igf2 *locus Nature 2000 405 486 489 10.1038/35013106 10839547

[B11] Kanduri C Pant V Loukinov D Pugacheva E Qi CF Wolffe A Ohlsson R Lobanenkov VV Functional association of CTCF with the insulator upstream of the *H19 *gene is parent of origin-specific and methylation-sensitive Curr Biol 2000 10 853 856 10.1016/S0960-9822(00)00597-2 10899010

[B12] Phillips JE Corces VG CTCF: master weaver of the genome Cell 2009 137 1194 1211 10.1016/j.cell.2009.06.001 19563753 PMC3040116

[B13] Parelho V Hadjur S Spivakov M Leleu M Sauer S Gregson HC Jarmuz A Canzonetta C Webster Z Nesterova T Cobb BS Yokomori K Dillon N Aragon L Fisher AG Merkenschlager M Cohesins functionally associate with CTCF on mammalian chromosome arms Cell 2008 132 422 433 10.1016/j.cell.2008.01.011 18237772

[B14] Rubio ED Reiss DJ Welcsh PL Disteche CM Filippova GN Baliga NS Aebersold R Ranish JA Krumm A CTCF physically links cohesin to chromatin Proc Natl Acad Sci USA 2008 105 8309 8314 10.1073/pnas.0801273105 18550811 PMC2448833

[B15] Stedman W Kang H Lin S Kissil JL Bartolomei MS Lieberman PM Cohesins localize with CTCF at the KSHV latency control region and at cellular c-myc and *H19*/*Igf2 *insulators EMBO J 2008 27 654 666 10.1038/emboj.2008.1 18219272 PMC2262040

[B16] Wendt KS Yoshida K Itoh T Bando M Koch B Schirghuber E Tsutsumi S Nagae G Ishihara K Mishiro T Yahata K Imamoto F Aburatani H Nakao M Imamoto N Maeshima K Shirahige K Peters JM Cohesin mediates transcriptional insulation by CCCTC-binding factor Nature 2008 451 796 801 10.1038/nature06634 18235444

[B17] Hadjur S Williams LM Ryan NK Cobb BS Sexton T Fraser P Fisher AG Merkenschlager M Cohesins form chromosomal *cis*-interactions at the developmentally regulated *IFNG *locus Nature 2009 460 410 413 19458616 10.1038/nature08079 PMC2869028

[B18] Hou C Dale R Dean A Cell type specificity of chromatin organization mediated by CTCF and cohesin Proc Natl Acad Sci USA 2010 107 3651 3656 10.1073/pnas.0912087107 20133600 PMC2840441

[B19] Nativio R Wendt KS Ito Y Huddleston JE Uribe-Lewis S Woodfine K Krueger C Reik W Peters JM Murrell A Cohesin is required for higher-order chromatin conformation at the imprinted *IGF2*-*H19 *locus PLoS Genet 2009 5 e1000739 10.1371/journal.pgen.1000739 19956766 PMC2776306

[B20] Kacem S Feil R Chromatin mechanisms in genomic imprinting Mamm Genome 2009 20 544 556 10.1007/s00335-009-9223-4 19760321

[B21] Wen B Wu H Bjornsson H Green RD Irizarry R Feinberg AP Overlapping euchromatin/heterochromatin-associated marks are enriched in imprinted gene regions and predict allele-specific modification Genome Res 2008 18 1806 1813 10.1101/gr.067587.108 18849526 PMC2577870

[B22] Diss TC Pan L Peng H Wotherspoon AC Isaacson PG Sources of DNA for detecting B cell monoclonality using PCR J Clin Pathol 1994 47 493 496 10.1136/jcp.47.6.493 8063927 PMC494724

[B23] Nielsen J Christiansen J Lykke-Andersen J Johnsen AH Wewer UM Nielsen FC A family of insulin-like growth factor II mRNA-binding proteins represses translation in late development Mol Cell Biol 1999 19 1262 1270 9891060 10.1128/mcb.19.2.1262 PMC116055

[B24] Noubissi FK Elcheva I Bhatia N Shakoori A Ougolkov A Liu J Minamoto T Ross J Fuchs SY Spiegelman VS CRD-BP mediates stabilization of *βTrCP1 *and c-*myc *mRNA in response to β-catenin signalling Nature 2006 441 898 901 10.1038/nature04839 16778892

[B25] Runge S Nielsen FC Nielsen J Lykke-Andersen J Wewer UM Christiansen J H19 RNA binds four molecules of insulin-like growth factor II mRNA-binding protein J Biol Chem 2000 275 29562 29569 10.1074/jbc.M001156200 10875929

[B26] Engel N Thorvaldsen JL Bartolomei MS CTCF binding sites promote transcription initiation and prevent DNA methylation on the maternal allele at the imprinted *H19*/*Igf2 *locus Hum Mol Genet 2006 15 2945 2954 10.1093/hmg/ddl237 16928784

[B27] Mahony S Hendrix D Golden A Smith TJ Rokhsar DS Transcription factor binding site identification using the self-organizing map Bioinformatics 2005 21 1807 1814 10.1093/bioinformatics/bti256 15647296

[B28] Mahony S Benos PV STAMP: a web tool for exploring DNA-binding motif similarities Nucleic Acids Res 2007 35 Web server W253 W258 10.1093/nar/gkm272 PMC1933206 17478497

[B29] Sandelin A Alkema W Engström P Wasserman WW Lenhard B JASPAR: an open-access database for eukaryotic transcription factor binding profiles Nucleic Acids Res 2004 32 Database D91 D94 10.1093/nar/gkh012 PMC308747 14681366

[B30] Kim TH Abdullaev ZK Smith AD Ching KA Loukinov DI Green RD Zhang MQ Lobanenkov VV Ren B Analysis of the vertebrate insulator protein CTCF-binding sites in the human genome Cell 2007 128 1231 1245 10.1016/j.cell.2006.12.048 17382889 PMC2572726

[B31] Zhang ZD Rozowsky J Snyder M Chang J Gerstein M Modeling ChIP sequencing in silico with applications PLoS Comput Biol 2008 4 e1000158 10.1371/journal.pcbi.1000158 18725927 PMC2507756

[B32] Gombert WM Krumm A Targeted deletion of multiple CTCF-binding elements in the human C-MYC gene reveals a requirement for CTCF in C-MYC expression PLoS One 2009 4 e6109 10.1371/journal.pone.0006109 19568426 PMC2699473

[B33] Bock C Reither S Mikeska T Paulsen M Walter J Lengauer T BiQ Analyzer: visualization and quality control for DNA methylation data from bisulfite sequencing Bioinformatics 2005 21 4067 4068 10.1093/bioinformatics/bti652 16141249

[B34] Guenther MG Levine SS Boyer LA Jaenisch R Young RA A chromatin landmark and transcription initiation at most promoters in human cells Cell 2007 130 77 88 10.1016/j.cell.2007.05.042 17632057 PMC3200295

[B35] Krumm A Hickey LB Groudine M Promoter-proximal pausing of RNA polymerase II defines a general rate-limiting step after transcription initiation Genes Dev 1995 9 559 572 10.1101/gad.9.5.559 7698646

[B36] O'Brien T Lis JT RNA polymerase II pauses at the 5' end of the transcriptionally induced *Drosophila **hsp70 *gene Mol Cell Biol 1991 11 5285 5290 1922045 10.1128/mcb.11.10.5285-5290.1991 PMC361584

[B37] Zeitlinger J Stark A Kellis M Hong JW Nechaev S Adelman K Levine M Young RA RNA polymerase stalling at developmental control genes in the *Drosophila melanogaster *embryo Nat Genet 2007 39 1512 1516 10.1038/ng.2007.26 17994019 PMC2824921

[B38] Heintzman ND Hon GC Hawkins RD Kheradpour P Stark A Harp LF Ye Z Lee LK Stuart RK Ching CW Ching KA Antosiewicz-Bourget JE Liu H Zhang X Green RD Lobanenkov VV Stewart R Thomson JA Crawford GE Kellis M Ren B Histone modifications at human enhancers reflect global cell-type-specific gene expression Nature 2009 459 108 112 10.1038/nature07829 19295514 PMC2910248

[B39] Mikkelsen TS Xu Z Zhang X Wang L Gimble JM Lander ES Rosen ED Comparative epigenomic analysis of murine and human adipogenesis Cell 2010 143 156 169 10.1016/j.cell.2010.09.006 20887899 PMC2950833

[B40] Renda M Baglivo I Burgess-Beusse B Esposito S Fattorusso R Felsenfeld G Pedone PV Critical DNA binding interactions of the insulator protein CTCF: a small number of zinc fingers mediate strong binding, and a single finger-DNA interaction controls binding at imprinted loci J Biol Chem 2007 282 33336 33345 10.1074/jbc.M706213200 17827499

[B41] Kadota M Yang HH Hu N Wang C Hu Y Taylor PR Buetow KH Lee MP Allele-specific chromatin immunoprecipitation studies show genetic influence on chromatin state in human genome PLoS Genet 2007 3 e81 10.1371/journal.pgen.0030081 17511522 PMC1868950

[B42] Kerkel K Spadola A Yuan E Kosek J Jiang L Hod E Li K Murty VV Schupf N Vilain E Morris M Haghighi F Tycko B Genomic surveys by methylation-sensitive SNP analysis identify sequence-dependent allele-specific DNA methylation Nat Genet 2008 40 904 908 10.1038/ng.174 18568024

[B43] Knight JC Keating BJ Rockett KA Kwiatkowski DP *In vivo *characterization of regulatory polymorphisms by allele-specific quantification of RNA polymerase loading Nat Genet 2003 33 469 475 10.1038/ng1124 12627232

[B44] Maynard ND Chen J Stuart RK Fan JB Ren B Genome-wide mapping of allele-specific protein-DNA interactions in human cells Nat Methods 2008 5 307 309 18345007 10.1038/nmeth.1194

[B45] McCann JA Muro EM Palmer C Palidwor G Porter CJ Andrade-Navarro MA Rudnicki MA ChIP on SNP-chip for genome-wide analysis of human histone H4 hyperacetylation BMC Genomics 2007 8 322 10.1186/1471-2164-8-322 17868463 PMC2194786

[B46] Delaval K Govin J Cerqueira F Rousseaux S Khochbin S Feil R Differential histone modifications mark mouse imprinting control regions during spermatogenesis EMBO J 2007 26 720 729 10.1038/sj.emboj.7601513 17255950 PMC1794379

[B47] Fournier C Goto Y Ballestar E Delaval K Hever AM Esteller M Feil R Allele-specific histone lysine methylation marks regulatory regions at imprinted mouse genes EMBO J 2002 21 6560 6570 10.1093/emboj/cdf655 12456662 PMC136958

[B48] Mikkelsen TS Ku M Jaffe DB Issac B Lieberman E Giannoukos G Alvarez P Brockman W Kim TK Koche RP Lee W Mendenhall E O'Donovan A Presser A Russ C Xie X Meissner A Wernig M Jaenisch R Nusbaum C Lander ES Bernstein BE Genome-wide maps of chromatin state in pluripotent and lineage-committed cells Nature 2007 448 553 560 10.1038/nature06008 17603471 PMC2921165

[B49] Ioannidis P Kottaridi C Dimitriadis E Courtis N Mahaira L Talieri M Giannopoulos A Iliadis K Papaioannou D Nasioulas G Trangas T Expression of the RNA-binding protein CRD-BP in brain and non-small cell lung tumors Cancer Lett 2004 209 245 250 10.1016/j.canlet.2003.12.015 15159028

[B50] Ioannidis P Mahaira L Papadopoulou A Teixeira MR Heim S Andersen JA Evangelou E Dafni U Pandis N Trangas T CRD-BP: a c-Myc mRNA stabilizing protein with an oncofetal pattern of expression Anticancer Res 2003 23 2179 2183 12894594

[B51] Ioannidis P Mahaira L Papadopoulou A Teixeira MR Heim S Andersen JA Evangelou E Dafni U Pandis N Trangas T 8q24 copy number gains and expression of the c-myc mRNA stabilizing protein *CRD-BP *in primary breast carcinomas Int J Cancer 2003 104 54 59 10.1002/ijc.10794 12532419

[B52] Ioannidis P Trangas T Dimitriadis E Samiotaki M Kyriazoglou I Tsiapalis CM Kittas C Agnantis N Nielsen FC Nielsen J Christiansen J Pandis N C-*MYC *and IGF-II mRNA-binding protein (CRD-BP/IMP-1) in benign and malignant mesenchymal tumors Int J Cancer 2001 94 480 484 10.1002/ijc.1512 11745432

[B53] Bieda M Xu X Singer MA Green R Farnham PJ Unbiased location analysis of E2F1-binding sites suggests a widespread role for E2F1 in the human genome Genome Res 2006 16 595 605 10.1101/gr.4887606 16606705 PMC1457046

[B54] Gombert WM Farris SD Rubio ED Morey-Rosler KM Schubach WH Krumm A The c-*myc *insulator element and matrix attachment regions define the c-*myc *chromosomal domain Mol Cell Biol 2003 23 9338 9348 10.1128/MCB.23.24.9338-9348.2003 14645543 PMC309672

[B55] Flanagin S Nelson JD Castner DG Denisenko O Bomsztyk K Microplate-based chromatin immunoprecipitation method, Matrix ChIP: a platform to study signaling of complex genomic events Nucleic Acids Res 2008 36 e17 18203739 10.1093/nar/gkn001 PMC2241906

[B56] Dignam JD Lebovitz RM Roeder RG Accurate transcription initiation by RNA polymerase II in a soluble extract from isolated mammalian nuclei Nucleic Acids Res 1983 11 1475 1489 10.1093/nar/11.5.1475 6828386 PMC325809

[B57] Chung JH Bell AC Felsenfeld G Characterization of the chicken β-globin insulator Proc Natl Acad Sci USA 1997 94 575 580 10.1073/pnas.94.2.575 9012826 PMC19555

