# Supplementary material for: Allele-specific transcriptional elongation regulates monoallelic expression of the IGF2BP1 gene
Source: Epigenetics Chromatin. 2011 Aug 3;4:14. doi: 10.1186/1756-8935-4-14 (PMC3174113; doi:10.1186/1756-8935-4-14)
Supplement: Additional file 1 — Table S1. Genomic coordinates of 293 genomic sites that are marked by both CTCF and H2K9me3. Table S2. List of genes tested for monoallelic expression in lymphoblastoid cell lines. [file 1756-8935-4-14-S1.DOC]

**Additional file 1**

Table S1: Coordinates of 293 genomic sites that are marked by both CTCF and H2K9me3 (Bedfile format for upload into the UCSC Genome Browser).

track name="CTCF3MK9 HBL100" description="3MK9CTCFGW"

chr1 477681 478031

chr1 584662 585912

chr1 11832651 11833301

chr1 16657242 16657692

chr1 26295094 26296013

chr1 27402552 27402902

chr1 36555469 36555919

chr1 55249202 55249652

chr1 110266194 110266644

chr1 110308542 110308892

chr1 120203323 120203673

chr1 142192377 142192827

chr1 142198676 142199026

chr1 142300667 142301017

chr1 142788648 142788998

chr1 143526793 143527143

chr1 145357132 145357482

chr1 150463325 150463675

chr1 154870372 154870822

chr1 157992823 157993173

chr1 197955350 197955800

chr1 198835838 198836388

chr1 200402859 200403309

chr1 200571947 200572297

chr1 202492338 202492688

chr1 222340217 222340867

chr1 226563359 226563709

chr10 13742015 13742465

chr10 15291843 15292293

chr10 70822039 70822589

chr10 71545851 71546301

chr10 102763905 102764241

chr10 103529601 103530452

chr10 104228740 104229490

chr10 105355360 105355710

chr10 131199200 131199750

chr10 134015466 134016416

chr11 1980005 1980455

chr11 3802641 3803091

chr11 45161192 45161542

chr11 47157121 47157671

chr11 61312088 61312438

chr11 62117631 62118181

chr11 63205903 63206253

chr11 63830086 63830836

chr11 64013725 64014075

chr11 64728740 64729190

chr11 65076143 65076493

chr11 65377130 65377480

chr11 65408732 65409182

chr11 66019464 66019814

chr11 116164289 116164639

chr11 120328578 120329034

chr12 432645 432995

chr12 6542815 6543265

chr12 47723675 47724225

chr12 50660492 50660842

chr12 51893362 51893912

chr12 53084366 53084716

chr12 55681532 55681882

chr12 55853312 55853662

chr12 105793414 105793852

chr12 108815335 108815685

chr12 112755581 112755931

chr12 118704681 118705231

chr12 126364902 126365252

chr12 128199483 128199933

chr12 129023251 129024056

chr12 131825712 131826162

chr13 23472787 23473137

chr13 32581878 32582328

chr13 98837662 98838112

chr13 110776563 110777113

chr13 113172906 113173656

chr14 23098413 23098863

chr14 23975790 23976240

chr14 68448201 68448551

chr14 73265359 73265662

chr14 74317859 74318509

chr14 76320799 76321149

chr14 76837129 76837479

chr14 76995337 76995687

chr14 92509288 92509638

chr14 99690661 99691211

chr14 104549643 104550193

chr14 104975322 104975672

chr14 105537473 105538223

chr14 105669489 105669939

chr14 105836157 105836607

chr14 105944495 105944945

chr14 106145593 106145943

chr14 106154004 106154454

chr14 106190922 106191372

chr14 106221847 106222297

chr15 18486664 18487014

chr15 18821682 18822132

chr15 19498053 19498403

chr15 19937505 19938155

chr15 19963009 19963759

chr15 20388368 20388706

chr15 24634752 24635402

chr15 38417486 38418000

chr15 57240809 57241159

chr15 63949125 63949775

chr15 70279062 70279412

chr15 71781902 71782252

chr15 71784202 71784552

chr15 78252887 78253237

chr15 80926291 80926641

chr15 82647519 82647869

chr15 88232593 88232943

chr15 88826317 88827267

chr15 100012784 100013534

chr16 2143524 2143974

chr16 2576169 2576719

chr16 2550816 2551166

chr16 13936896 13937246

chr16 18455777 18456127

chr16 32858746 32859096

chr16 32901352 32902303

chr16 33623504 33623955

chr16 33623502 33623852

chr16 33671479 33671929

chr16 65331231 65331781

chr16 66475016 66475466

chr16 66519795 66520245

chr16 68858903 68859253

chr16 86566596 86567146

chr17 16533832 16534282

chr17 24101380 24101930

chr17 30349158 30349708

chr17 34144981 34145531

chr17 37099691 37100041

chr17 39313320 39313770

chr17 39783579 39784029

chr17 40529082 40529432

chr17 40703875 40704225

chr17 43410834 43411184

chr17 44468326 44468676

chr17 45511494 45512244

chr17 45572320 45572670

chr17 52546499 52546849

chr17 69233986 69234636

chr17 71160830 71161180

chr17 71355927 71356377

chr17 71434850 71435500

chr17 71905467 71906217

chr17 74500920 74501270

chr17 76818761 76819211

chr17 77787604 77787954

chr18 75613522 75614172

chr19 4517917 4518467

chr19 15224279 15224829

chr19 16389729 16390079

chr19 17697278 17697628

chr19 18441060 18441610

chr19 48053204 48053654

chr19 48375631 48376081

chr19 48394847 48395197

chr19 48458513 48459063

chr19 50254399 50254749

chr19 51923854 51924204

chr19 54226853 54227203

chr19 54231722 54232072

chr19 54292807 54293257

chr19 55682054 55682404

chr19 59552758 59553308

chr2 3168480 3168830

chr2 6862264 6862914

chr2 24962191 24962541

chr2 24991121 24991571

chr2 27512455 27513205

chr2 73032321 73032671

chr2 85908464 85908792

chr2 96848765 96849315

chr2 101113285 101113735

chr2 171527328 171527878

chr2 191686678 191687028

chr2 217796919 217797269

chr2 219338814 219339164

chr2 219692427 219693177

chr2 219987918 219988568

chr2 220211395 220212245

chr2 233136370 233136820

chr2 236437155 236437505

chr2 236656678 236657128

chr2 238363606 238364056

chr2 242238987 242239537

chr20 29620951 29621301

chr20 30841558 30842008

chr20 33053216 33053766

chr20 33355027 33355477

chr20 35446552 35447002

chr20 36399540 36399890

chr20 47938456 47938806

chr20 48979239 48979689

chr20 60413374 60413824

chr20 60770344 60770794

chr21 44358280 44358730

chr22 19246981 19247331

chr22 21000815 21001165

chr22 21957136 21957586

chr22 29992914 29993992

chr22 35231578 35232028

chr22 36343939 36344289

chr22 38234508 38235158

chr22 39966678 39967228

chr22 40238266 40238716

chr22 48946918 48947968

chr22 48950636 48951086

chr22 49016392 49017542

chr3 13433109 13433459

chr3 33148135 33148485

chr3 45945442 45945792

chr3 49026316 49026766

chr3 50269609 50269959

chr3 50336899 50337260

chr3 51401849 51402299

chr3 52300467 52300817

chr3 52480889 52481239

chr3 120347009 120347459

chr3 139270024 139270474

chr3 140246582 140246932

chr3 188940376 188940926

chr3 196991445 196991995

chr4 641562 642112

chr4 1302123 1302573

chr4 2993750 2994500

chr4 109309084 109309434

chr4 187884751 187885401

chr5 722307 722657

chr5 1564784 1565134

chr5 3814151 3815201

chr5 14404961 14405311

chr5 16509371 16509721

chr5 140734980 140735330

chr5 141505180 141505530

chr5 150408295 150408645

chr5 180796878 180797328

chr6 546269 546619

chr6 7284302 7284652

chr6 11152877 11153227

chr6 18230712 18231223

chr6 33355618 33356168

chr6 43848969 43849519

chr6 169380895 169381445

chr6 170939788 170940238

chr7 560157 560707

chr7 599554 600004

chr7 1750654 1751204

chr7 5839210 5839760

chr7 23228057 23228507

chr7 44198481 44198931

chr7 45534581 45535131

chr7 63087604 63087954

chr7 65466613 65466963

chr7 65746086 65746436

chr7 73260251 73260701

chr7 74692504 74692954

chr7 142519354 142519904

chr8 442944 443394

chr8 653717 654967

chr8 141418021 141418371

chr8 145222370 145223520

chr9 988558 989008

chr9 33456451 33457201

chr9 33912215 33912565

chr9 34242931 34243381

chr9 43570996 43571346

chr9 64252609 64252959

chr9 45426423 45426773

chr9 66874784 66875134

chr9 67113609 67114159

chr9 89507461 89507811

chr9 92476354 92476804

chr9 93434230 93434580

chr9 120281044 120281494

chr9 126240366 126240716

chr9 127784414 127785064

chr9 128973483 128973933

chr9 128984903 128985253

chr9 129952001 129952351

chr9 131083160 131083610

chr9 131383209 131383559

chr9 133248477 133248927

chr9 133298618 133299068

chr9 134517202 134517552

chr9 135622383 135622733

chr9 136896851 136897201

chr9 137234234 137234684

chr9 138283608 138284258

chrX 153122403 153122753

chrY 2540108 2540458

Table S2

| **Gene name** | **Fraction of heterozygous individuals** | **cDNA Analysis** |
| --- | --- | --- |
| IGF2BP1 | 10/15 | 6 Monoallelic |
| DIAPH1 | 3/13 | Biallelic |
| FUS1 | 4/8 | Biallelic |
| PKP1 | 7/8 | Biallelic |
| ARFGAP2 | 5/9 | Biallelic |
| PCDHGA | 2/7 | Biallelic |
| MTHFR | 4/8 | Biallelic |
| LAIR1 | 6/11 | Biallelic |
| GPR3 | 0/13 | N/A |
| ARMET | 0/13 | N/A |
| NPR1 | 0/13 | N/A |
| NHLRC1 | 0/13 | N/A |
