# Supplementary material for: Allele-specific transcriptional elongation regulates monoallelic expression of the IGF2BP1 gene
Source: Epigenetics Chromatin. 2011 Aug 3;4:14. doi: 10.1186/1756-8935-4-14 (PMC3174113; doi:10.1186/1756-8935-4-14)
Supplement: Additional file 2 — Figure S1. Detection and colocalization of CTCF and H3K9me3 at the human IGF2-H19 ICR locus by ChIP-chip experiments. Top: Enrichment of CTCF binding sites. Middle: Results of large-scale array-based chromatin immunoprecipitation (ChIP-chip) survey of histone H3 trimethylated at lysine 9 (H3K9me3) binding. Bottom: H19 exons demonstrating positions of CTCF binding and histone modifications relative to exons. Figure S2. Analysis of the clonal status of lymphoblastoid cell lines used in this study. Following the protocol described in [22], PCR amplification of two regions within the variable segment in the immunoglobulin heavy chain gene (conserved framework region 2 (Fr2) and the variable joining regions (VLJH)) reveals the clonal status of lymphoblastoid cell lines (LCLs). The amplification product from a polyclonal population (P) gives rise to fragments of varying length due to the large number of rearranged immunoglobulin genes and appears as a broad band. Amplification of DNA derived from monoclonal cell lines results in one or two discrete bands within an expected size range of 240 to 280 bp. The polyclonal sample (P) was obtained from the peripheral blood of a healthy donor. Lanes 1 through 4: monoclonal cell lines GM7007, GM7033, GM6989 and GM7030. Lanes 5 through 8: monoclonal lines GM7050, GM7023, GM7059 and GM7057. MW, DNA size marker. Figure S3. Sequencing results give results identical to those derived from the TaqMan allelic discrimination assay. (A) Standard sequencing results of two individuals at SNP site rs9904288. (B) TaqMan allelic discrimination assay confirms the heterozygosity of GM7057 and the homozygosity of GM6990. Figure S4. Quantitative assessment of TaqMan genotyping using specific probe set at SNP rs11655950. The 3'-UTR of the IGF2BP1 gene was amplified using primers given in Supplemental Table 2. This segment contains an A/G SNP. The PCRs included a FAM-labeled probe for the A allele and a VIC-labeled probe for the B allele. After PCR amp [file 1756-8935-4-14-S2.DOC]

# Additional File 2

**
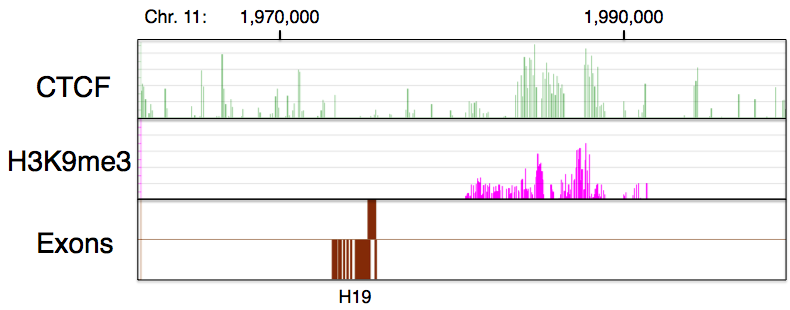
**

**Figure S1**


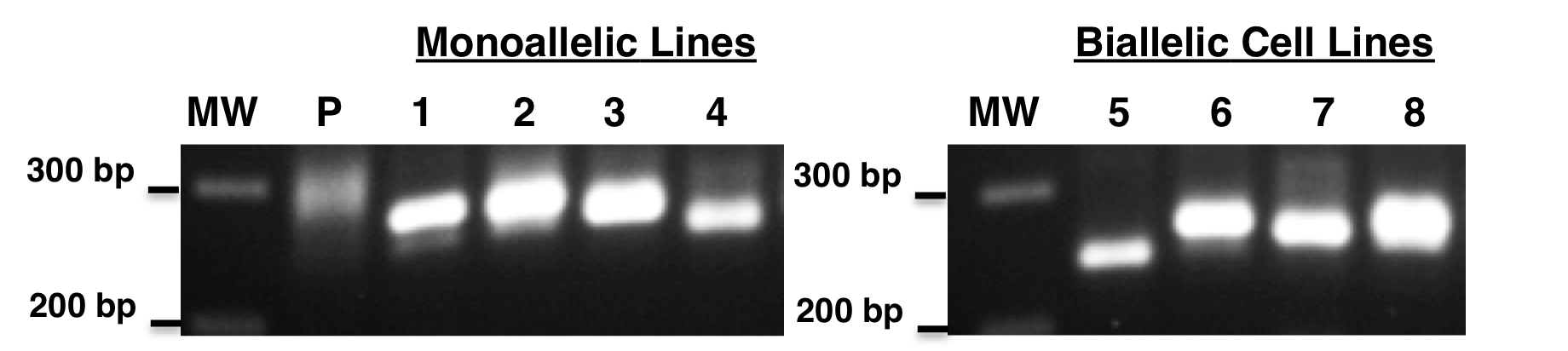


**Figure S2**


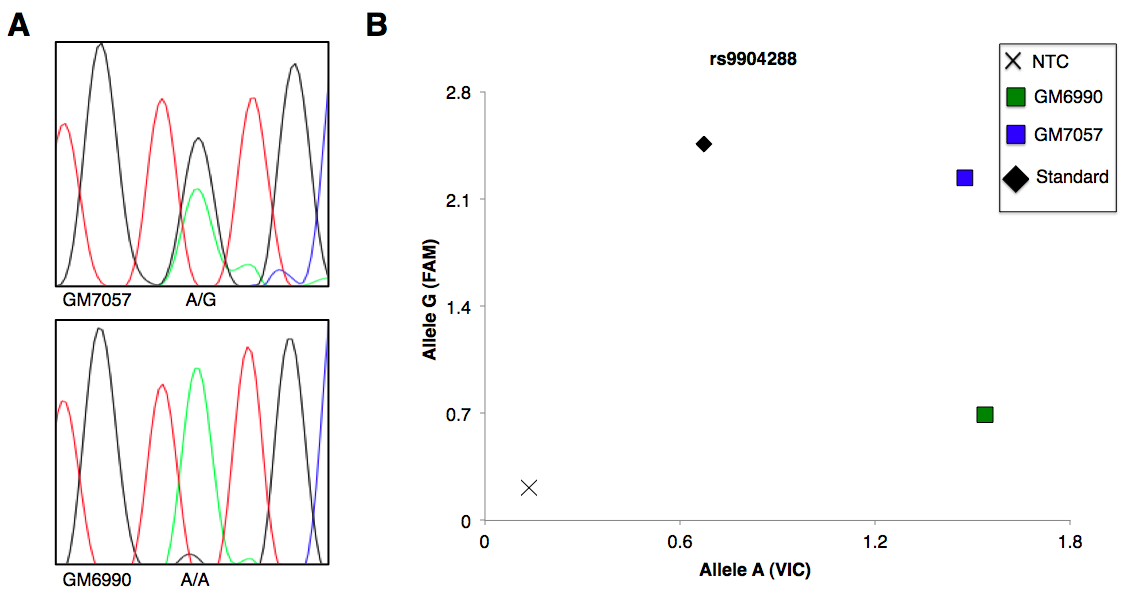


**Figure S3**


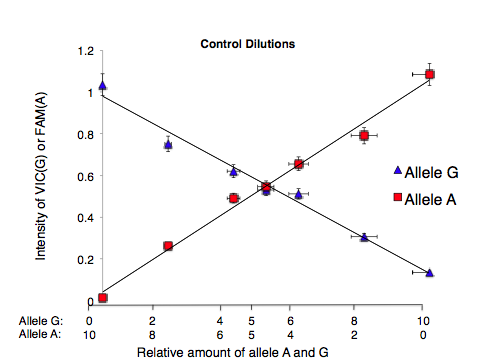


**Figure S4**


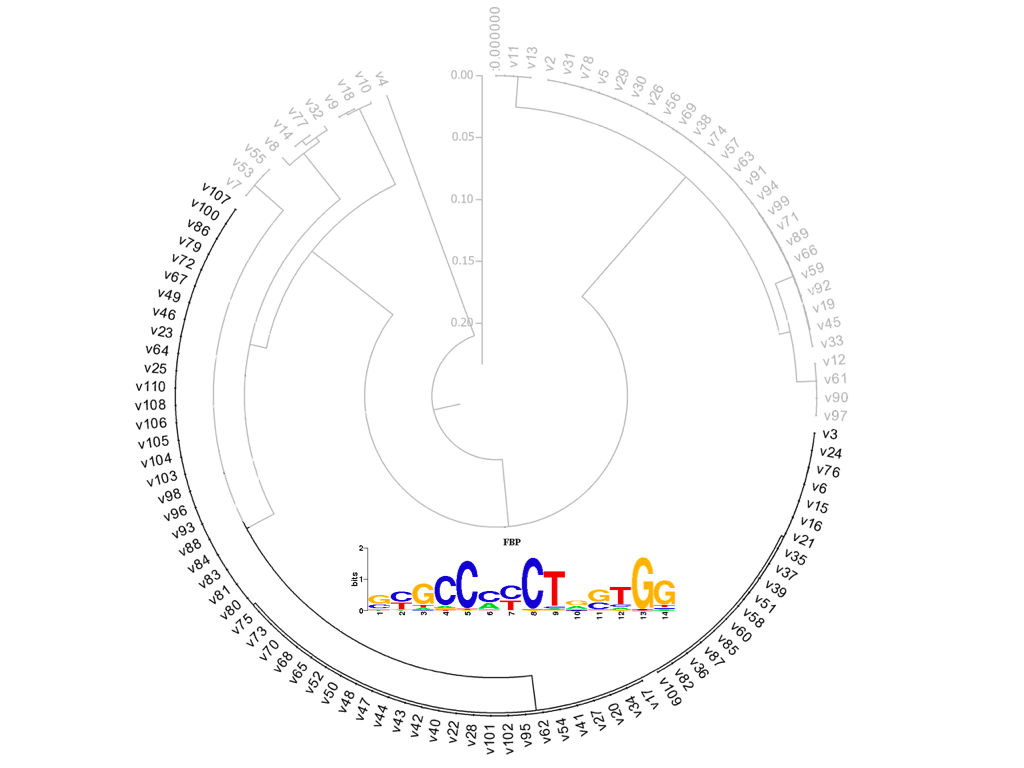


**Figure S5**


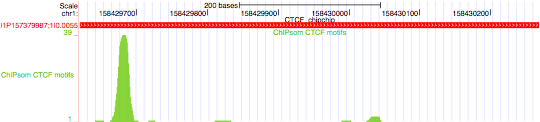


**Figure S6**


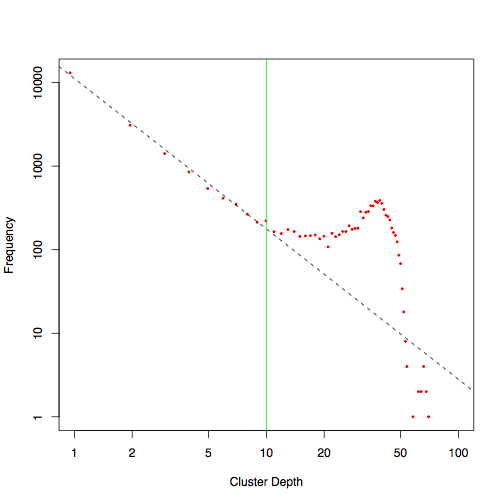


**Figure S7**


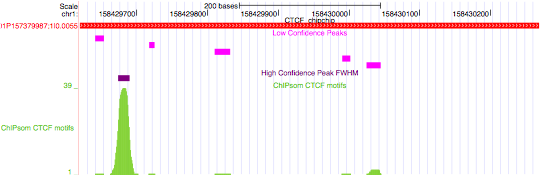


**Figure S8**

.

Y^wt^ 105

CTTGGCAAGTATATAGCAGGTAGTAAAATGGCTGTAATTTTGAAAGTTAGCTTTGTGAAGTGTTGGGGCTGCGGCATCTGCTGGTGGTGTGTGGTATTACAGCCC

Y^mut^ 105

CTTGGCAAGTATATAGCAGGTAGTAAAATGGCTGTAATTTTGAAAGTTAGCTTTGTGAAGTGTTGGGGCTGCGGCATTTGTTATTGGTGTGTGGTATTACAGCCC

## Y^wt^ Z^wt^ 125

CTTGGCAAGTATATAGCAGGTAGTAAAATGGCTGTAATTTTGAAAGTTAGCTTTGTGAAGTGTTGGGGCTGCGGCATCTGCTGGTGGTGTGTGGTATTACAGCCCCGTGCTGGCGGACCCCATTG

Y^wt^ Z^mut^ 125

CTTGGCAAGTATATAGCAGGTAGTAAAATGGCTGTAATTTTGAAAGTTAGCTTTGTGAAGTGTTGGGGCTGCGGCATCTGCTGGTGGTGTGTGGTATTACAtgCatGTGCTatCGaACtCCATTG

Y^mut^Z^wt^ 125

CTTGGCAAGTATATAGCAGGTAGTAAAATGGCTGTAATTTTGAAAGTTAGCTTTGTGAAGTGTTGGGGCTGCGGCATTTGTTATTGGTGTGTGGTATTACAGCCCCGTGCTGGCGGACCCCATTG

Y^mut^Z^mut^ 125

CTTGGCAAGTATATAGCAGGTAGTAAAATGGCTGTAATTTTGAAAGTTAGCTTTGTGAAGTGTTGGGGCTGCGGCATTTGTTATTGGTGTGTGGTATTACAtgCatGTGCTatCGaACtCCATTG

Y^mut^ chFII 105

CTTGGCAAGTATATAGCAGGTAGTAAAATGGCTGTAATTTTGAAAGTTAGCTTTGTGAAGTGTTGGGGCTGCGGCATCTGGCGGTGGTGTGTGGTATTACAGCCC

Y^mut^ mmR3 105

CTTGGCAAGTATATAGCAGGTAGTAAAATGGCTGTAATTTTGAAAGTTAGCTTTGTGAAGTGTTGGGGCTGCGCCACCGCGCGGTGGTGTGTGGTATTACAGCCC

IGF2 wt huB1

TGTGATGTGTGAGCCTGCACTGCCGCCGCGCGGCCACTTCCGATTCCACAACT

# Figure S9
